# Rhizarthrosis Part I: A Literature Review

**DOI:** 10.7759/cureus.52932

**Published:** 2024-01-25

**Authors:** Saverio Colonna, Corrado Borghi

**Affiliations:** 1 Osteopathic Spine Center Education (OSCE), Spine Center, Bologna, ITA

**Keywords:** rhizarthrosis, biomechanics, dynamic stability, therapeutic exercise, mobilization, manual therapy, trapezium-metacarpal joint

## Abstract

Rhizarthrosis (RA), or trapeziometacarpal osteoarthritis, is an arthritic degenerative process that affects the first joint of the thumb. The objective of this work is to provide therapists with an overview of the fundamental issues related to the therapeutic management of trapeziometacarpal joint instability.

Prevalent in females, especially post-menopause, and linked to age, RA involves ligament and muscle structures, with causes ranging from hormonal influences to mechanical factors. Understanding the biomechanics, stability, and factors contributing to RA is crucial for effective intervention. This study explores the role of ligaments, muscles, and anatomical variants in thumb joint degeneration, emphasizing the importance of stability and congruence.

RA manifests as pain at the base of the thumb, limiting grip strength and hindering everyday tasks. Pain initially occurs during specific movements but can progress to constant discomfort, affecting sleep. Chronic RA leads to joint stiffness, deformities like the "Z thumb," and muscle atrophy, impacting daily functions.

Clinical evaluation involves pain assessment, joint mobility examination, and palpation. Diagnostic tests like the grind test and lever test aid in confirming RA. Radiographic examination reveals joint space degeneration and osteophytes and helps classify RA stages using the Eaton-Littler classification.

Conservative treatment aims to alleviate pain, reduce joint stress, and enhance function. Orthoses help stabilize the joint. Therapeutic exercises, emphasizing muscle strength and dynamic stability, prove beneficial. Manual therapies like neurodynamic, Kaltenborn, Mulligan, and Maitland techniques target pain reduction and improve joint mechanics.

The studies on conservative approaches provide evidence that a multimodal intervention consisting of joint mobilization, neural mobilization, and exercise is beneficial in reducing pain in patients with RA. When conservative therapy fails, surgical intervention is indicated.

## Introduction and background

**Figure 1:**
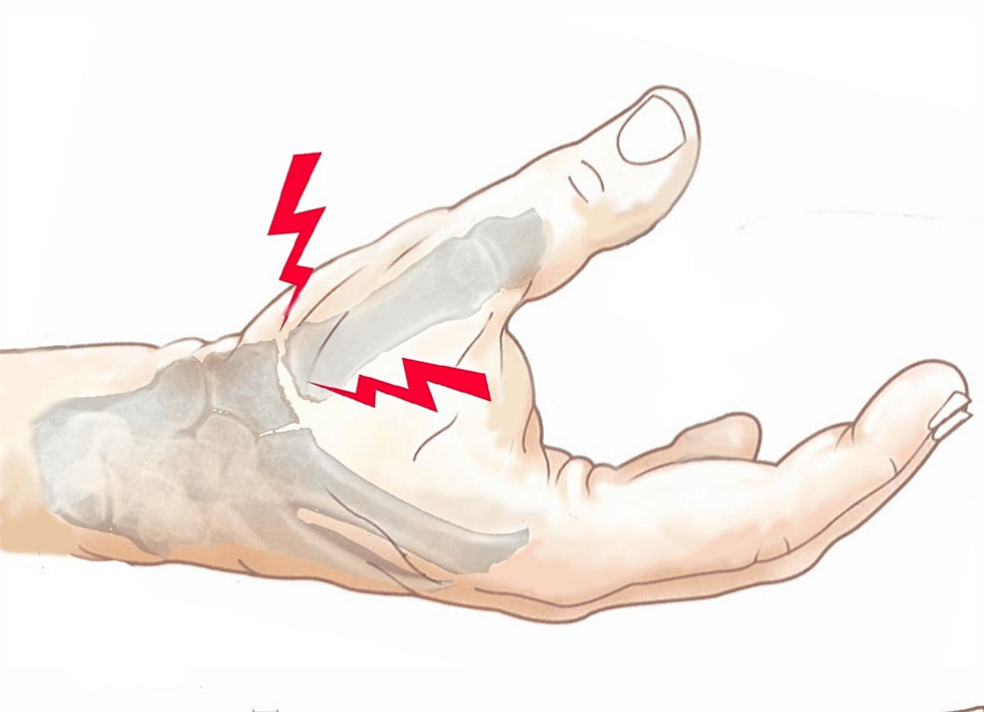
Trapeziometacarpal joint (TMj).

Rhizarthrosis (RA), or trapeziometacarpal osteoarthritis, is an arthritic degenerative process that affects the first joint of the thumb, i.e., the one between the trapezium bone and the base of the first metacarpal bone (Figure 1). The thumb joint is a joint whose biomechanics is characterized by the need for multidimensional mobility that allows rotational movements, also called circling movements [1-3]. The thumb is responsible for more than 40% of the functions of the hand because the ability to grasp and squeeze is ineffective without its opposability and its prehensile abilities [4]. Therefore, a degeneration of the thumb joint can be very disabling.

In a comparative radioclinical study on osteoarthritis (OA), which involved 592 subjects aged 50 years or over, radiographs of 32 joints were performed to evaluate the prevalence and radiological localization of OA, finding that the carpometacarpal and metacarpophalangeal joints of the thumb are among the most affected joints [5]. RA is more prevalent in females compared to males (in patients over 35 years, the incidence is, respectively, 15% and 1.4%). The risk in both sexes increases with age, reaching 25% of women after menopause [6], while more than half of the cases are of women over 71 years [7]. This greater predisposition of women for this pathology can be attributed to a greater laxity present in females and according to Ateshian et al. [8], to the different congruence in the two sexes: the concavity of the metacarpal surface and the convexity of the trapezoidal surface are less pronounced in women than in men. Other authors, more recently, found no difference between the two sexes. Instead, they reported that age has the main role in reducing congruence and predisposing to RA [9].

**Figure 2:**
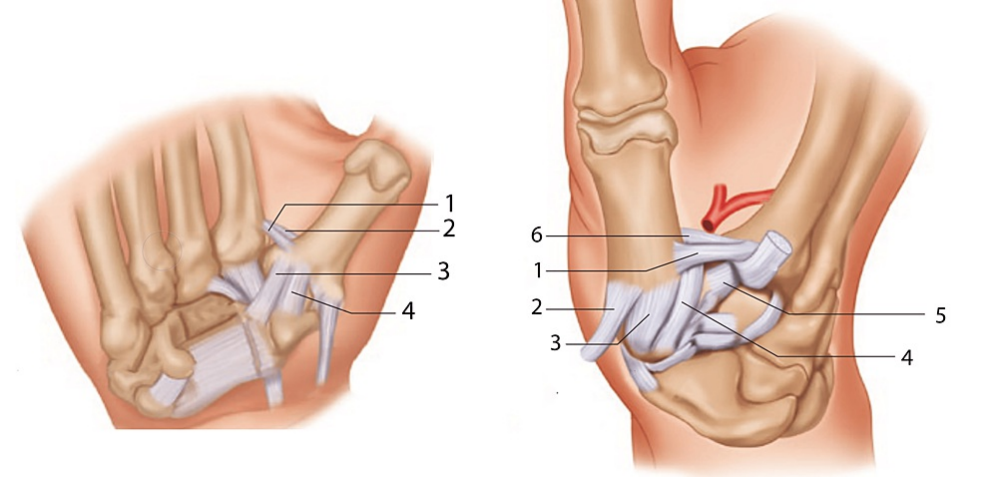
Ligamentous structures of the trapeziometacarpal joint. Superficial volar ligaments on the left: 1 - ventral intermetacarpal; 2 - dorsal intermetacarpal; 3 - ulnar collateral; 4 - superficial anterior oblique). Dorsal view on the right: 1 - dorsal intermetacarpal; 2 - abductor pollicis longus; 3 - dorsoradial; 4 - posterior oblique; 5 - dorsal trapezio–second metacarpal; 6 - ventral intermetacarpal. Figure adapted from [11]. Used with permission from Springer Nature.

The stability of this joint (trapeziometacarpal joint - TMj) is due to the fine balance between the passive ligament structures and the active muscle-tendon structures. The passive stability of the TMj is mainly determined by five ligaments (Figure 2) [10,11]: anterior oblique divided into superficial and deep (beak ligament); ulnar collateral; inter metacarpal; posterior oblique; and radial collateral. Although there is controversy over the primary passive stabilizers, several studies have concluded that the anterior oblique ligament and radial collateral, also known as the dorsal radial, are the primary stabilizers [12,13].

**Figure 3:**
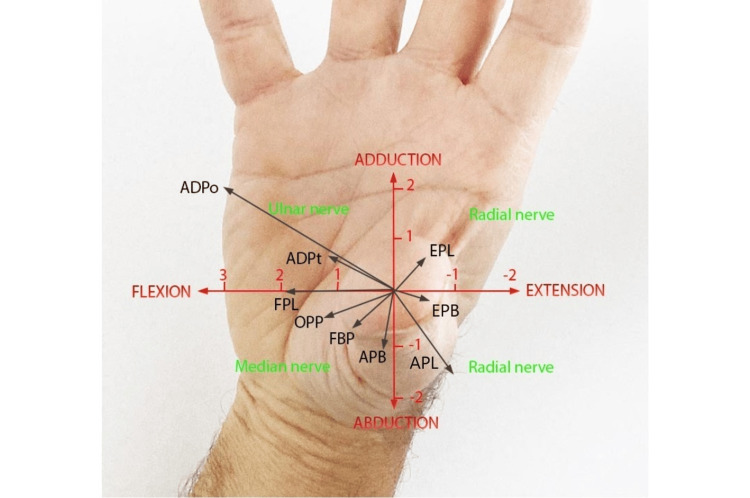
Graphical representation of the muscles' action on the trapeziometacarpal joint. The direction of muscle pull is indicated by the orientation of the lines, while the torque potential is proportional to their length (indicated in Nm on the flex-extension/ab-adduction plane). The torque potential was obtained by considering both the cross-section and the lever arm. ADPo: adductor of the thumb, oblique head; ADPt: adductor of the thumb, transverse head; OPP: opponent of the thumb; FBP: short flexor of the thumb; APB: abductor short of the thumb; APL: long abductor of the thumb; EPB: short extensor of the thumb; EPL: long extensor of the thumb. The diagram is based on data originally plotted by Smutz et al. [17].

The muscles that have a relevant action on the TMj are adductor pollicis (oblique and transverse head), short and long flexor (deep and superficial head), short and long abductor, opponent, short and long extensor, first palmar and dorsal interossei, and lumbricals [14]. Active stability, managed by the thumb muscles, has not yet been clarified [15]. The activation of each muscle provides a force vector, which, on the one hand, can improve congruity and, on the other, could increase instability. Figure 3 shows the direction of the force vector of most of the muscles that act on the TMj [10]. Regarding the compaction force determined by the muscles during the grip, a dated study by Cooney and Chao showed that during a grip that generates 1 kg at the level of the fingertips, the calculated compression loads were 3 kg at the interphalangeal joint, 5.4 kg at the metacarpal phalangeal joint, and 12 kg at the TMj [16].

The causes of TMj degeneration are not precisely known. The high incidence in women over 50 years suggests there is a strong hormonal involvement [6]. However, as reported above, other causes, like mechanical factors, are reasonably present.

**Figure 4:**
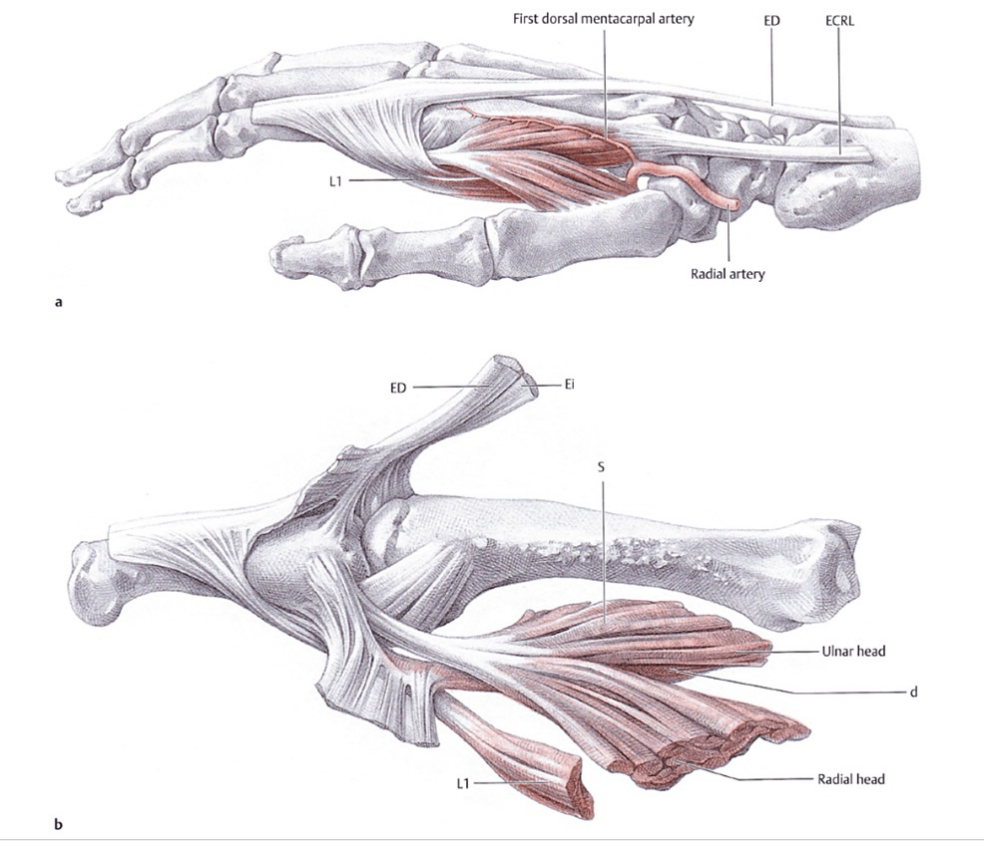
Anatomy of the first dorsal interosseous. (a) Muscles in place; (b) details of origins and insertions. ED: extensor digitorum; Ei: extensor indicis; ECRL: extensor carpi radialis longus; L1: first lumbricals; S: 1st DI superficial layer; d: 1st DI deep layer. Adapted from Schmidt and Lanz [20]. Used with permission from Thieme.

The first dorsal interosseous (1st DI) has been recognized as critical in the stability of the TMj by the biomechanical studies of Brand and Hollister [18]: when in the lateral pinch position with the thumb muscles loaded, if the tension from 1st DI was removed, TMj had a radial subluxation; after the tension of this muscle was restored, the joint repositioned itself correctly. Boutan identified a torque effect of the 1st DI and the opponent on the base of the 1st metacarpus and found that the 1st DI is a very active thumb muscle during closed kinetic chain prehensile activities [19]. The 1st DI is a muscle with a dual proximal insertion: the radial one occurs on the proximal half of the first metacarpus, and the ulnar one on the second metacarpus; the distal insertion is realized with a single tendon at the base of the proximal phalanx of the second finger (Figure 4).

**Figure 5:**
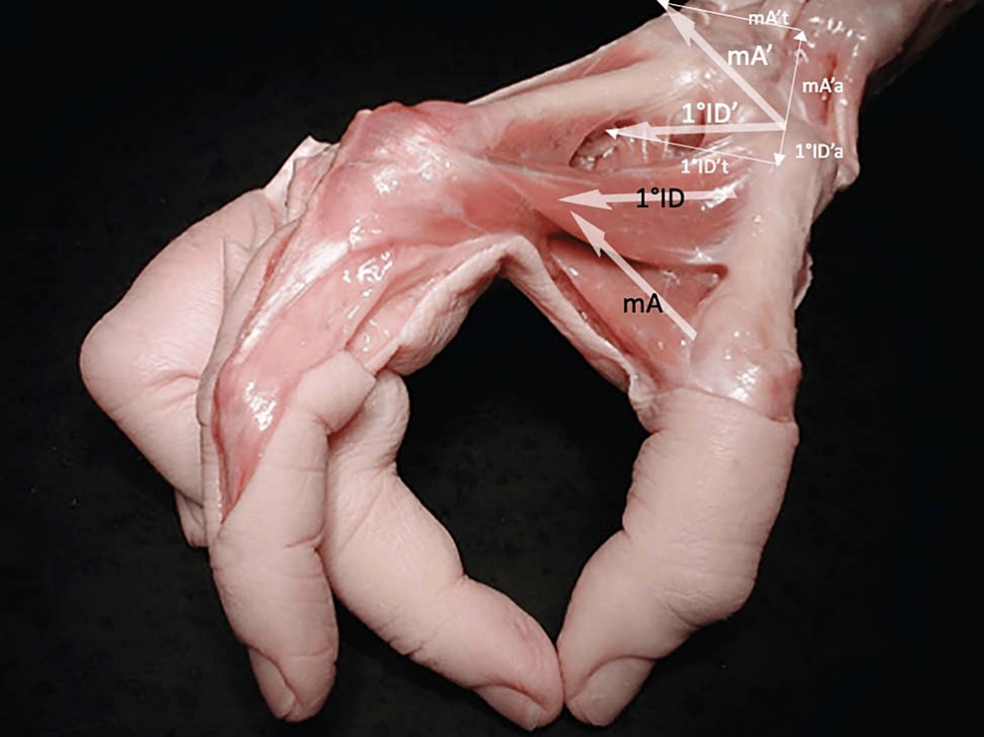
Force vectors during gripping. The relationship of the force vectors during gripping with the 1st DI and the adductor muscle (mA) is highlighted. Translation of vectors at the level of the TMj: 1st DI’ and mA'. Breakdown of the forces in the two vectors: mA’t and 1st DI’t transversal component; mA'a and 1st DI axial component. TMj: trapeziometacarpal joint; 1st DI: first dorsal interosseous. Modified from Schmidt and Lanz [20]. Used with permission from Thieme.

The 1st DI is innervated by the deep branch of the ulnar nerve [21]. An electromyographic study on the activation of the hand muscles, in particular of the 1st DI, recognized that RA decreases strength and increases the execution time of dexterity tasks [22]. Moulton et al. identified greater TMj congruence when the metacarpal is flexed at 30° relative to the trapezius, with less load on the volar surface [23]. In this position, on one hand, the vectors of the oblique head of the adductor muscle and opponent muscle, and, on the other hand, the vector of the 1st DI, have the same direction (agonists) on the transverse plane (plane orthogonal to the major axis of the metacarpus) in bringing the first metacarpus closer to the second; however, on the main axis of the TMj, they have opposite directions (antagonists). This implies that the action of the oblique head of the adductor and opponent (indirectly also the flexor) leads to radial compression/sliding of the proximal epiphysis (dislocation), while the 1st DI is opposed (Figure 5).

**Figure 6:**
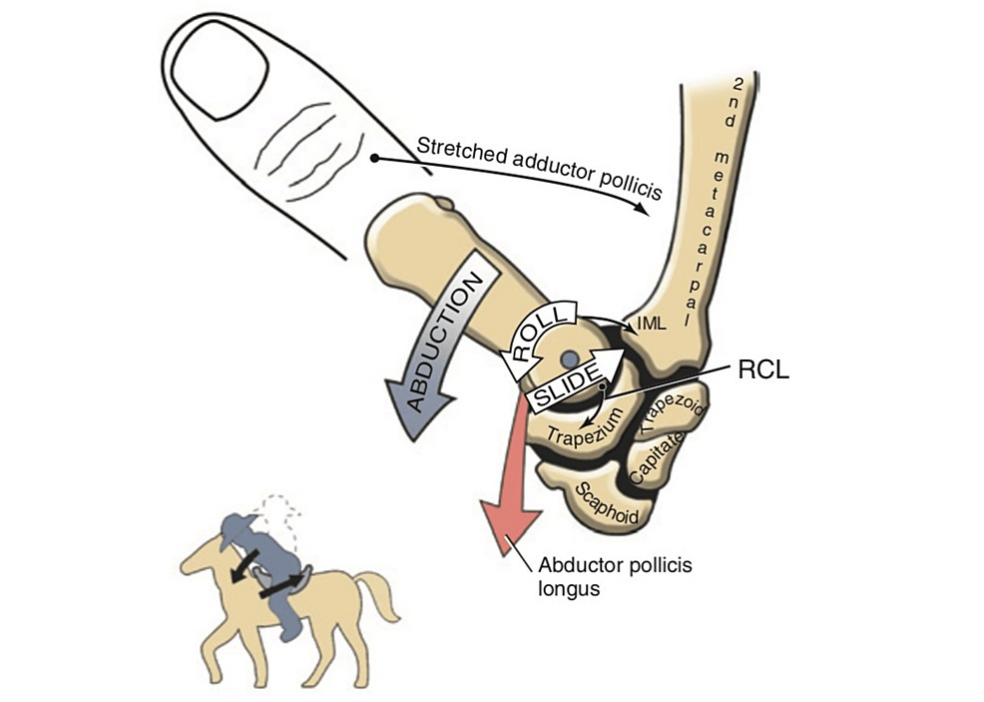
Arthrokinematics of trapeziometacarpal joint abduction. The axis of rotation is represented by the small circle at the base of the metacarpus. IML represents the intermetacarpal ligament, and RCL represents the radial collateral ligament. Note the analogy of abduction and the cowboy falling forward on the horse's saddle: the chest "rotates" forward, and the pelvis "slides" backward. Adapted from Neumann et al. [14]. Used with permission from Elsevier.

**Figure 7:**
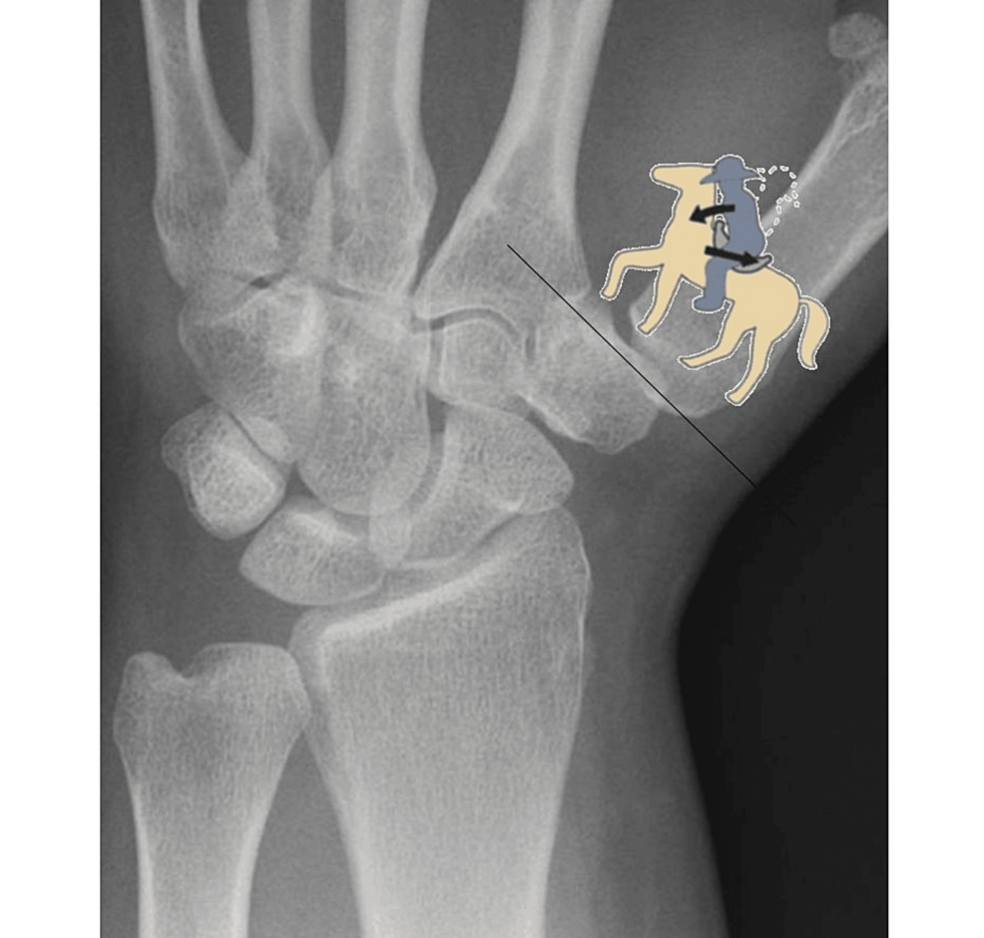
The direction of the rider is more in accordance with the movements and stresses of the trapeziometacarpal joint.

**Figure 8:**
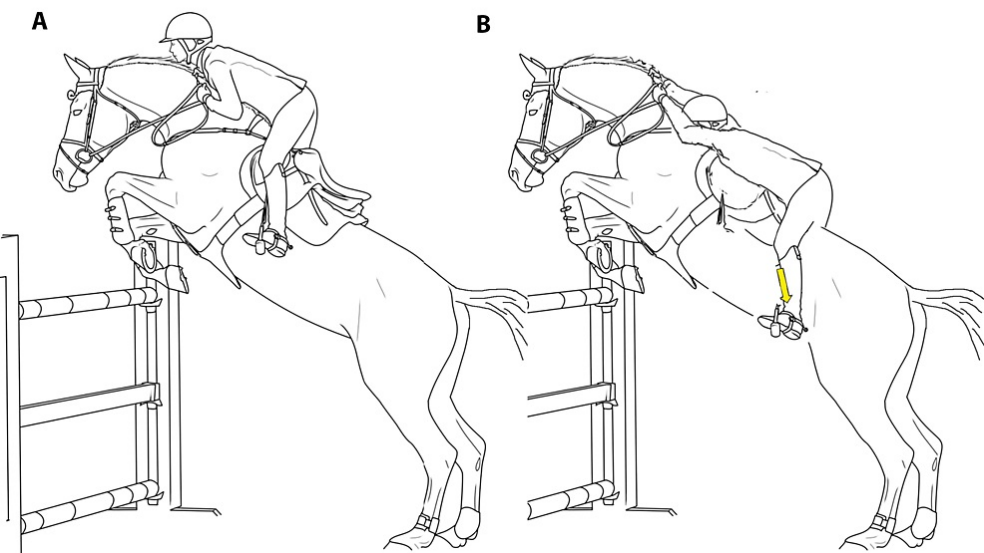
Trapeziometacarpal joint and stirrups. The imbalance of the jockey during the jump (with broken stirrups, B) gives the idea of the important function of the stirrups (A).

Considering the image of the horse and the rider proposed by Neumann (Figure 6), to better understand the imbalance at the base of the RA, it is necessary to reconsider first the direction of the rider and second the "horse racing gesture" [14]. About the direction, the ulnar direction (face facing the 2nd metacarpus) is closer to reality (Figure 7) than the radial one; moreover, the equestrian gesture of jumping at obstacles is more suitable to describe RA causes than the horse racing gesture. In this case, the orientation of the saddle is more inclined, and the rider must seek balance. During the jump, the rider needs the stirrups to keep stability (Figure 8A); in the event of a break in the stirrups, the rider would rotate forward and slide backward (Figure 8B). The stirrups are representative of the ligamentous structures. RA, therefore, is often secondary to insufficient ligament structures.

Laxity, especially of the anterior oblique ligament of the TMj, causes instability of the joint during translational movements [24]. It is believed that, due to this insufficiency of the anterior oblique ligament, the resulting recurrent stresses during the movements of the first column of the thumb are at the basis of the degeneration of the cartilage [15], which begins on the palmar surface and then extend over the dorsoradial one [25,26].

Some professions, which involve greater manual activity, are more easily subject to the development of RA due to the greater stress to which TMj is subjected. There are also familiar forms or forms associated with hypermobility that gradually lead to OA of TMj. RA can also be secondary to trauma, or a constitutional hyperlaxity, as in some connective tissue diseases, such as Marfan disease [27] and Ehlers-Danlos disease [28]. Sometimes OA can follow inflammatory rheumatism such as rheumatoid arthritis [29] or chondrocalcinosis [30]. Some anatomical variants of the trapezius, particularly when it is flat or oblique, by increasing joint instability, favor the onset of TMj OA [31].

## Review

Symptoms

**Figure 9:**
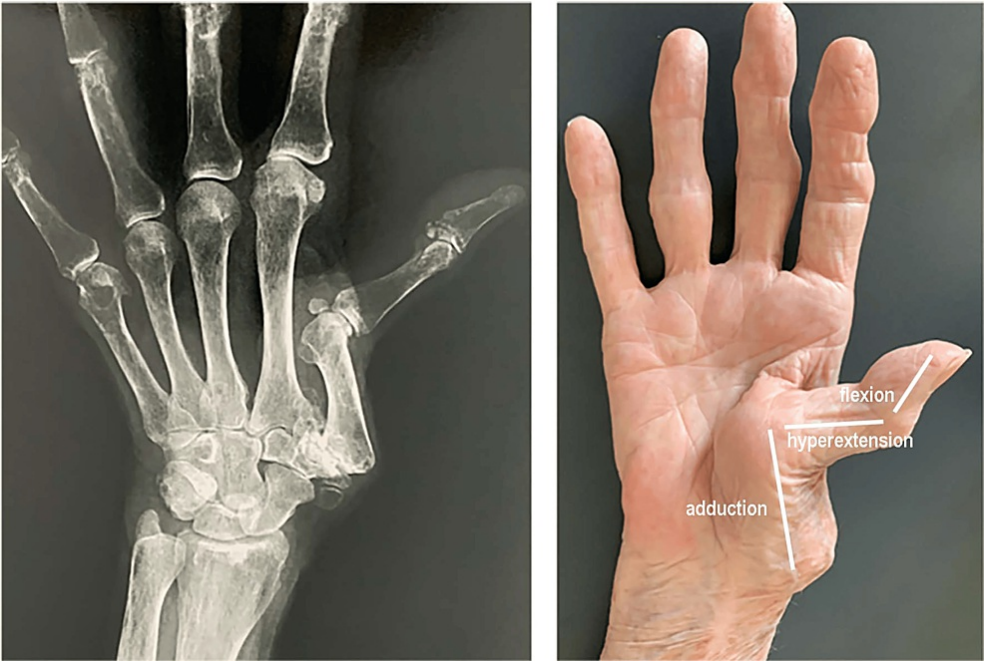
Z-thumb. Example of Z-strain of advanced rhizarthrosis. Modified from [35]. With courtesy of G Chick MD, PhD.

The pain due to RA is most often a pain located at the base of the thumb in the TMj. The patient may complain of decreased thumb-finger grip strength and an inability to grasp heavier or larger objects [32]. While in the initial phase, the pain is evoked mainly during mechanical stress and the execution of certain gestures, over time, it tends to manifest itself for any simple movement of the thumb up to present itself in the most serious cases even in conditions of rest, even during sleep [32]. The anatomical alteration of the joint leads to high joint stiffness, especially in the extension/abduction movement of the thumb. This rigidity becomes chronic over time also due to the fact that the person tends to immobilize the area to limit the evocation of pain; however, this leads to an ever-increasing functional limitation and therefore difficulty in performing manual gestures that require the use of the thumb [33]. In the most advanced stage, the thumb tends to deform into the so-called "Z thumb," which presents itself with an adduction of the first metacarpal on the trapezius bone, a compensatory hyperextension of the first phalanx on the first metacarpal, and a subsequent flexion of the second phalanx on the first phalanx (Figure 9) [34]. Chronic RA conditions often lead to hypotrophy of the thenar eminence musculature. Although RA usually manifests unilaterally at the beginning, often on the dominant side, over time the involvement of the other side is not infrequent.

Diagnosis

The diagnosis of RA is based initially on the qualitative assessment of the history, where attention is focused on the location, duration, onset, frequency, intensity, and quality of pain. Physical examination with the addition of special tests is important; later, complementary tests, such as X-rays, can be performed, if necessary [36,37].

Manual Evaluation

A review of the literature reports 52 clinical tests for the evaluation of TMj OA, which were classified according to five dimensions recognized as pillars of thumb stability and mobility: architecture, ligaments, biomechanics, neuromuscular control, and proprioception [38]. Despite a large number of assessments, the information provided remains insufficient regarding the quantification of ligamentous laxity, performance under load, individual thumb muscle strength, joint stability, and feed-forward control, which are needed to answer questions related to therapeutic intervention efficacy.

The clinical examination begins with the search for pain at the base of the first metacarpal under mechanical stress and palpation, thus also investigating any alterations in joint mobility and tissue changes. In palpation and the induction of movement of the first metacarpal bone on the trapezius, joint cracks and a strong rigidity are sometimes evident. At the positional and palpatory level, the metacarpal bone is strongly adducted on the axis of the hand [37].

**Figure 10:**
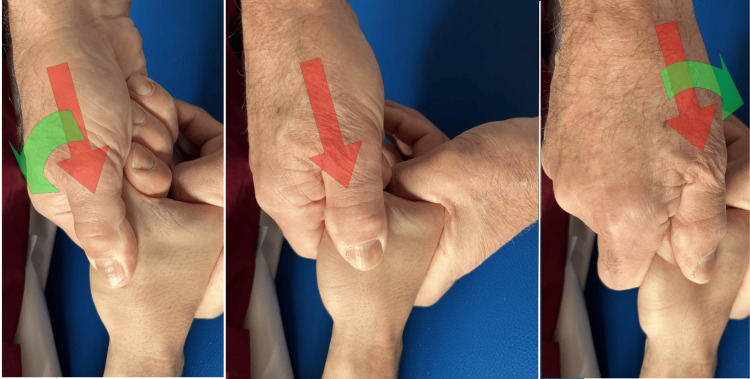
Grind test. The evaluation is carried out by compressing along the axis of the metacarpal bone and simultaneously rotating the metacarpal base of the thumb [41].

**Figure 11:**
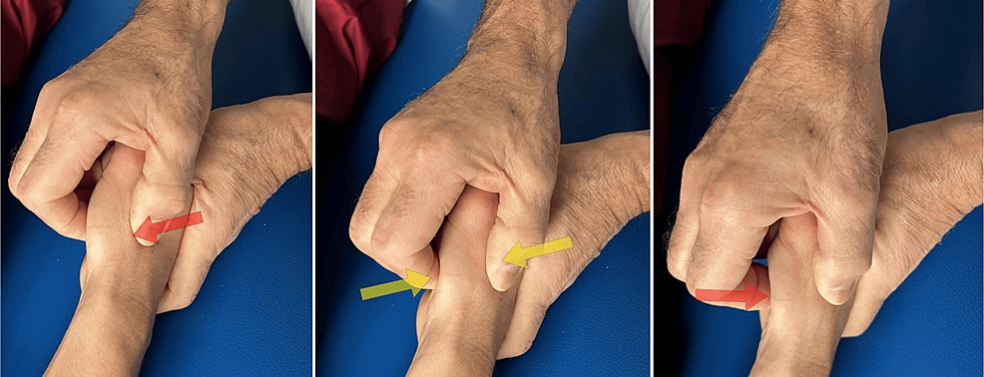
Lever test. The assessment is performed by placing the index finger and thumb on both sides of the TMj and exerting leverage on the TMj in both the radial and ulnar directions [41]. TMj: trapeziometacarpal joint.

Tests can be carried out to diagnose RA. The grind test is performed by firmly grasping the proximal phalanx of the thumb, pushing it toward the TMj, while making light rotational movements (Figure 10). A low radio-clinical correspondence between the grind test result and X-rays (weak kappa test, 0.46) was observed in a study of 59 subjects [39]. If the positive predictive value of the test is good (91%), the negative predictive value is low (68%), i.e., the absence of pain during the execution of this test does not exclude the radiographic diagnosis of metacarpophalangeal osteoarthritis. More recent studies involving a control group of healthy subjects confirmed this diagnostic finding [40]. Another test proposed in the literature is the lever test [41]. The lever test is performed by holding the first metacarpal distally just beyond the TMj with the index finger and thumb and moving it alternately in an ulnar and radial direction (Figure 11). The lever test is the test that produces the greatest pain, the pain most similar to that reported by the patient, and which also has the highest specificity and sensitivity. The grind test has high specificity but the lowest sensitivity [41]. Furthermore, the same authors consider direct palpation of the TMj, mainly on the palmar side, as a valid provocation test, capable of confirming the origin of the pain with greater accuracy. This latter test becomes important when differentiating RA pain from abductor brevis/extensor pollicis brevis tenosynovitis (de Quervain's disease).

Patients with painful TMj will also need to have a differential diagnosis for several other pathologies, such as scaphoid fracture, flexor carpi radialis tendinitis, carpal instability, synovial cyst, synovitis, and carpal tunnel [42]. Therefore, diagnostic tests are important for differential diagnosis (Table 1).

**Table 1:** Manual tests for differential diagnosis.

Pathology	Specific test
Scaphoid fractures	Snuffbox and scaphoid tubercle tenderness [43]
De Quervain's disease	Finkelstein's and Eichhoff's test [44]
Flexor carpi radialis tendinitis	Tenderness over volar radial forearm along flexor carpi radialis tendon. Provocative tests: (1) resisted wrist flexion triggers pain; (2) resisted radial wrist deviation triggers pain [45]
Carpal tunnel syndrome	Phalen’s and reverse Phalen’s test; carpal compression Durkan’s test; Tinel’s test [46]
Carpal instability	Dorsal intercalated segment instability (DISI) tests, volar intercalated segment instability (VISI) tests, and carpal instability nondissociative (CIND) tests [47]
Wrist synovial cyst	Diagnosed clinically based on history and examination findings [48]

Instrumental Evaluation

**Figure 12:**
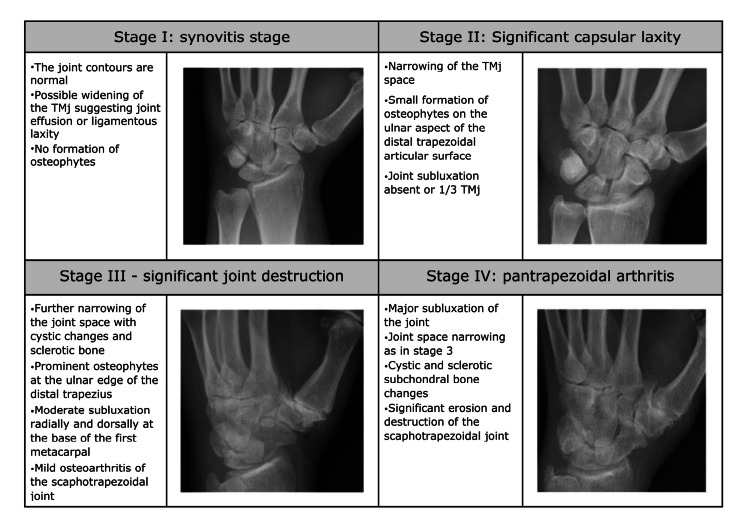
Stages of rhizarthrosis according to the Eaton-Littler classification. Stages (A) I, (B) II, (C) III, and (D) IV carpometacarpal osteoarthritis of the Eaton-Littler [49]. The descriptions are accompanied by original images. TMj: trapeziometacarpal joint.

Observing the X-rays, the patient with RA presents the disappearance or degeneration of the joint space between the base of the metacarpal and the trapezius, and the presence of osteophytes (or bone beaks) in the external perimeter of the joint. The stages of RA are generally classified according to the Eaton-Littler classification, which is based on radiological or arthroscopic procedures [49]. This classification is a staging protocol on four different stages based on synovitis, joint space, and capsule laxity (Figure 12).

Some authors affirm that there is no direct correlation between the level of joint involvement and the patient's clinical condition [50,51]. Ribak et al. find a correlation only in Eaton-Littler stages III and IV [37].

Conservative therapy

Conservative treatment essentially aims to reduce pain and mechanical load on the joint and relieve inflammation, thus maintaining or increasing strength, function, stability, and mobility while improving occupational performance. This treatment is preferred to surgery in mild forms at the initial stages, i.e., Eaton stages I and II of the disease [49,52]. Anyway, the choice of treatment depends more on the severity of the reported symptoms and the functional needs of the patient, than Eaton's radiographic staging.

Conservative treatment involves multiple therapeutic approaches: orthoses; therapeutic exercise; manual therapy; physical therapy; and infiltrative therapy.

Orthoses

The use of orthoses is the most used treatment in non-surgical forms [53]. The aims of using a splint on the TMj involve enhancing stability, alleviating pain, lowering inflammation, enhancing function, and lessening the mechanical stress that might trigger instability. Regrettably, wearing a splint merely offers external backing to the joint; once taken off, it ceases to deliver therapeutic benefits [10].

**Figure 13:**
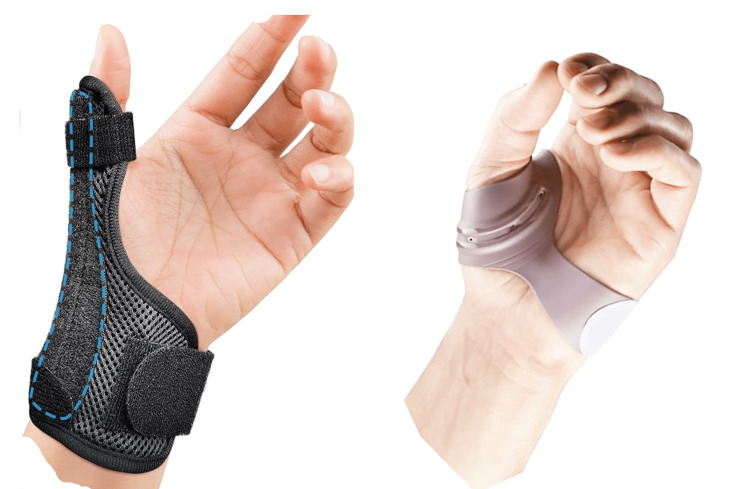
Examples of two models of splints for rhizarthrosis: long model on the left, and short model on the right.

Orthoses can delay the progression of the disease, by constraining the thumb in a condition of "forced rest" (Figure 13). They differ in materials and structure. The main two types are for static night use (wrist is included) or for functional diurnal use. Splinting keeps the thumb abducted and opposite to the 3rd finger (the patient is asked to reproduce an “O” during preparation), the metacarpophalangeal slightly flexed, and the interphalangeal of the thumb and long fingers free. This position allows to center the base of the trapezius on the 1st metacarpus, permitting correct joint positioning to limit the most painful and stressful extension and adduction movements for the joint. The research analyzed in a literature review delved into the effectiveness of static splinting in addressing primary TMj instability [54]. Weiss and colleagues conducted a study comparing the effectiveness of the long opponent splint with the less restrictive TMj immobilization splint [55]. Both splints showed comparable pain reduction, yet neither led to an improvement in pinch strength or a decrease in pain experienced during pinching actions. Orthoses use is associated with a targeted occupational therapy program that consists of gestural training and the possible use of aids with long lever arms that require less muscular effort.

As regards the conservative treatment of RA through the use of Kinesio tape, two studies report good results, one associating it with therapeutic exercise [56] and one with manual therapy [57].

Therapeutic Exercise

The thumb, known for its remarkable mobility among hand joints, relies on dynamic stability supported by the combined strength of muscles, bones, and ligaments. According to Poole and Pellegrini, reinforcing the thenar muscles, specifically the long abductor and the long extensor, is beneficial in preserving the dynamic stability of the thumb's basal joint complex [58].

The literature suggests a set of five exercises within an exercise program: thumb opposition, tearing paper, tracing a line on a ball's surface, employing sticks to grasp objects, and performing ball squeezes [53]. This exercise program aims to enhance thumb movement range (opposition and drawing lines on the ball), increase neuromuscular control of thumb alignment and muscle endurance (tearing paper and ball squeezing), and refine the thumb base joint's proprioception. Emphasis is placed on executing the exercises to prevent the collapse or overextension of the TMj while maintaining proper abduction. These exercises are grounded in recent evidence highlighting the significance of proprioception training and strengthening the 1st DI, aiming for practicality by simulating daily activities [59]. A comprehensive exercise guide is available visually through a website developed by physiotherapists affiliated with the New South Wales Department of Health in Sydney, Australia [60]. The strategy centered on dynamic stability involves several components: restoring thumb web space, re-educating extrinsic and intrinsic thumb muscles, with a focus on the 1st DI and its antagonists (thumb abductors and extensors), mobilizing the joint to manage pain, and strengthening the muscles responsible for upholding joint stability. Valdes and von der Heyde propose a series of exercises to be performed both actively and passively: thumb flexion, thumb adduction, thumb opposition, thumb TMj extension, and thumb interphalangeal flexion [61]. For OA of the hands, an additional protocol is proposed, consisting of a group of six exercises, but it is not specific for TMj [62]. A recent specific protocol for the treatment of OA proposes the strengthening of the first dorsal interosseous muscle. This protocol, still awaiting experimental verification, suggests the execution of six mobility and six strengthening exercises [63].

Manual Therapy

The authors who published the most on manual therapy applied to RA are Villafañe and colleagues [57,64-66]. These authors, in the four works mentioned above, proposed four different manual therapy techniques: neurodynamic (Butler), Kaltenborn, Mulligan, and Maitland.

**Figure 14:**
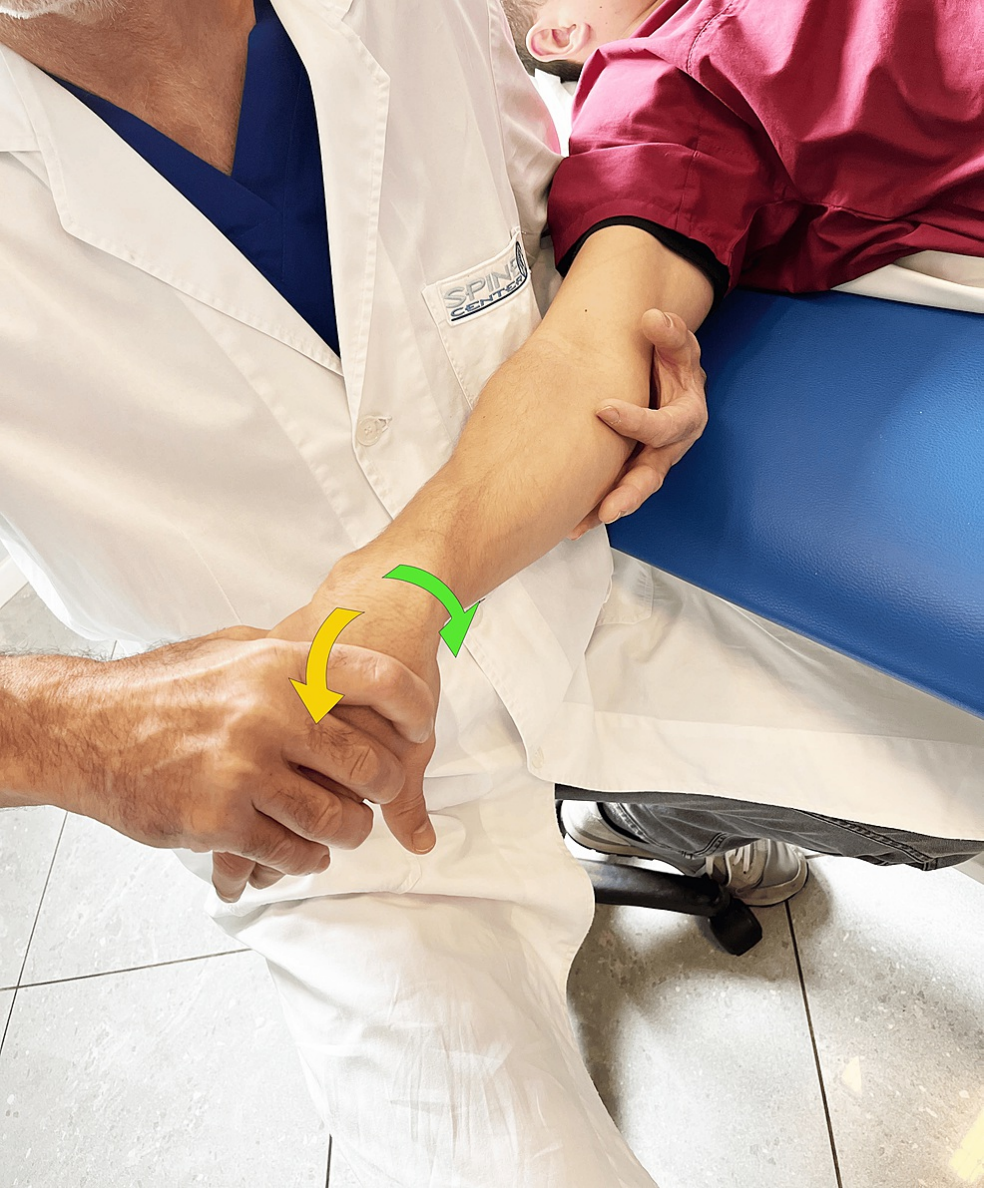
Neurodynamic technique. The technique of mobilization of the radial nerve with an experimental sliding technique [65].

Neurodynamic technique: Pain stands out as a primary symptom in individuals dealing with OA, often exacerbated by sensitization, that intensifies its severity. OA pain seems closely linked to localized degenerative changes like cartilage destruction, synovial inflammation, and bone alterations [67]. Interestingly, the intensity of pain does not consistently correlate with the extent of joint damage or inflammation, hinting at a possible central aspect of the pain experience [68]. In a study where 48 knee OA patients were analyzed, lowered pain thresholds under pressure were noted in both affected joints, along with heightened temporal summation compared to 24 healthy subjects [69]. The reported pain levels seemed linked to the degree of sensitization rather than observable radiological changes, suggesting a role for central sensitization in OA pain. Moreover, research by Bjordal et al. indicated that long-term use of non-steroidal anti-inflammatory drugs or acetaminophen did not significantly reduce pain levels in OA [70]. Similar findings of sensitization, both peripheral and central, were observed by Farrell et al. in knee OA patients, detecting mechanical, electrical, and thermal hyperalgesia around the TMj [71]. Neurodynamic techniques target neural structures by manipulating the position and movement of multiple joints [72]. The literature outlines two approaches for the application of these techniques: sliding and tensioning [72]. Sliding involves coordinated movements of at least two joints, wherein one movement increases nerve tension while the other releases it, offering a cyclical loading and unloading of the nerve [73]. Using gliding techniques for neurodynamic techniques proved to be highly effective. Conversely, tensioning techniques, which stress the nerve, may trigger abnormal mechanosensitive impulses [74], potentially leading to sustained heightened internal pressure that could impact intraneural blood flow [75]. The work of Villafañe et al. reports, in the treatment of TMj, a better result in 30 treated subjects compared to 30 control subjects, in terms of pain at direct pressure, in the tip and tripod pinch, using neurodynamic treatment [65]. The technique used in the above work (Figure 14) is as follows: the patient lying on his back; a physiotherapist, seated beside the limb being treated, applies pressure to lower the patient's shoulder while extending the elbow and internally rotating the arm; the patient's wrist, thumb, and all fingers are flexed; the hand is positioned in ulnar deviation [65]. This sequence of movements is intended to target and stress the radial nerve [72]. Once the upper extremity is positioned, two sets of movements are executed as follows: shoulder depression is performed simultaneously with elbow flexion and wrist extension; then, shoulder elevation is carried out with elbow extension, wrist flexion, and ulnar deviation. These movements have to be alternated at a speed of approximately two seconds per cycle, comprising one second in extension and one second in flexion.

**Figure 15:**
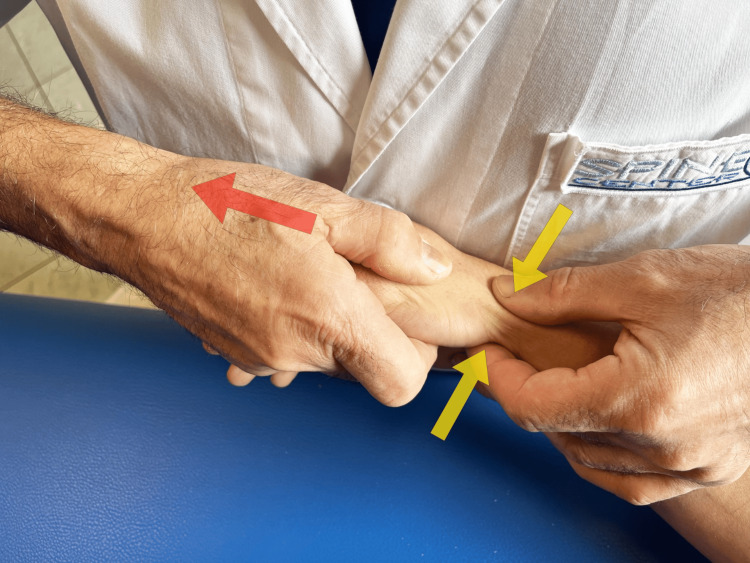
Kaltenborn technique. Trapeziometacarpal joint mobilizations according to the Kaltenborn concept [64].

Kaltenborn technique: The specific mobilization of Kaltenborn is the anteroposterior sliding with TMj grade 3 distraction [76]. In short, Kaltenborn and Evjenth described these mechanisms in terms of the convex-concave rule; the appropriate treatment can be inferred from this rule by following the direction of the decrease in joint sliding in a hypomobile joint. It is believed that the shape of the articular surface induces its gliding/sliding movement; a female (concave) joint surface slides in the same direction movement, while a male (convex) surface slides in the opposite direction to the bone movement [76,77]. Traction is the technique that distracts an articular surface perpendicularly to the other, while the sliding technique describes the translational sliding of one joint surface parallel to the other [78]. Grade 3 traction was defined as an additional force, which is applied on the parallel axis. This results in the stretching of the joint and the surrounding soft tissues, separating the articular surfaces [78]. The technique used in the reported work (Figure 15) consisted in [64] the patient being seated with the arm positioned anatomically, the elbow flexed at a 90-degree angle, and the hand and forearm facing downward with the back of the hand against the physiotherapist's body. Using the right thumb and forefinger, the physiotherapist takes the metacarpal bone of the subject's right thumb, initiating a gliding mobilization of short amplitude in the anteroposterior direction along with a distraction of the TMj. This mobilization is repeated for three minutes, followed by a one-minute break; the action is repeated three times. In the anteroposterior sliding movement of the first metacarpal bone, the head and body of the metacarpal must slide in the same way because the articular surface of the trapezius is convex, and the surface of the first metacarpal bone is concave; in this way, the sliding movement respects the rule of the convexity-concavity of the joint according to Kaltenborn and Evjenth [76].

**Figure 16:**
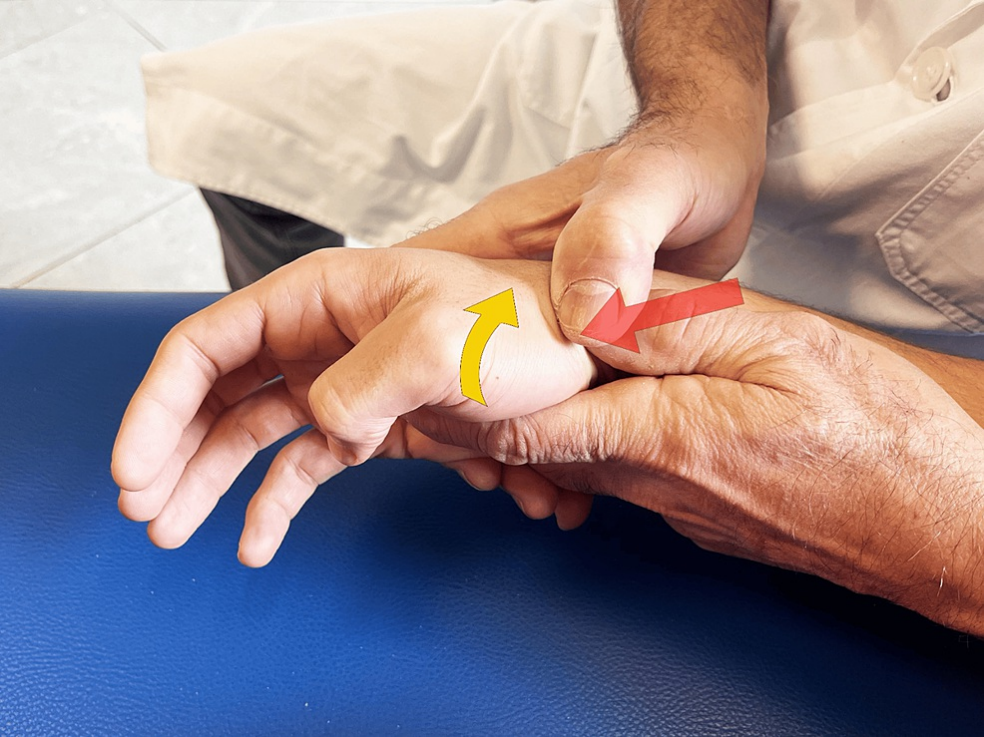
Mulligan technique. Trapeziometacarpal joint mobilizations according to the Mulligan concept [57].

Mulligan technique: Mulligan has developed and taught the unique "mobilization with movement" approach for joint dysfunction [79]. The principle behind this method suggests that even a slight misalignment or minor irregularity in joint positioning could lead to pain and limited movement. By manually guiding a painless adjustment of the joint while the patient performs a natural movement in the direction that was previously painful, the mechanics of the joint can be modified, reducing dysfunction. In contrast to the previously discussed joint mobilizations, which typically adhere to the biomechanical principle of the convex/concave rule [76], Mulligan's approach demonstrates that aiding an additional movement at the appropriate angle relative to the movement plane can generate a pain-free therapeutic effect, theoretically enhancing the joint's functional movement. This method relies on the absence of pain during the procedure as an indicator of its suitability. Seeing an improvement in the range of motion indicates that the technique applied is correct. Research and case studies show the effectiveness of mobilization with movement for both peripheral and central joints experiencing pain due to traumatic or non-traumatic joint disorders in physical therapy treatments. The technique used in the work reported above involves (Figure 16) the following [57]: initial evaluation, the active movements (radial and palmar TMj abduction and adduction) of the thumb limited by pain. To find the position that best allows pain-free movement, an iterative process is used. The therapist fine-tunes the direction and pressure to be imposed in the counterthrust applied by both thumbs on the base of the first metacarpal using the patient's feedback. With this action, the proximal epiphysis of the first metacarpus should be positioned in a more neutral condition by reducing the subluxation. This action improves the patient's ability to move the joint with less pain in previously painful angles. When adequately positioned, with the correct direction and strength of thumb mobilization, the patient performs three sets of 10 repetitions of each of the previously painful thumb movements, especially in the abduction/adduction plane.

**Figure 17:**
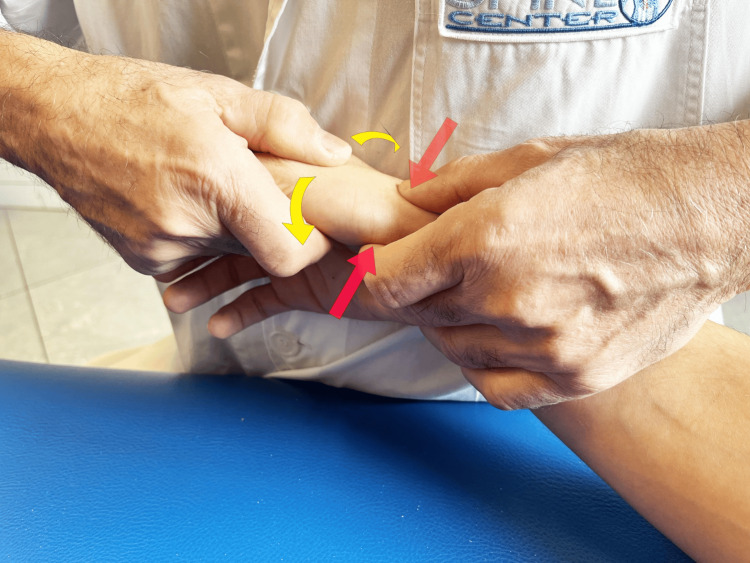
Maitland technique. Maitland's passive accessory mobilization technique: mobilization of posterior-anterior gliding of the trapeziometacarpal joint [66].

Maitland technique: A research study investigated the impact of Maitland's passive accessory mobilization on local hypoalgesia and strength in RA [66]. The treatment involved four sessions spread across two weeks, where patients received either four sessions of Maitland's passive accessory mobilization or a sham treatment for their dominant hand. This method included a specific mobilization technique known as anteroposterior gliding of the TMj (Figure 17). During the treatment, subjects sat with their arm in an anatomical position, their elbow bent at 90°, their hand and forearm with the cubital facing downwards, and the dorsal face of their hand against the physiotherapist's body. The physiotherapist, with thumb and forefinger on the metacarpal bone of the patient’s thumb, then performed specific passive accessory mobilization by applying anteroposterior gliding to the TMj, following Maitland's approach, for three minutes with a one-minute break, repeated three times [66]. The mobilization had to be performed by gentle small-amplitude oscillations, with the head and body of the metacarpal bone that slide in the same direction. To ensure no pain or discomfort occurred during the mobilization, the force applied was adjusted based on the subjects' feedback. The technique was performed within a comfortable range of motion. Approximately 60 oscillations per minute were conducted during the three-minute mobilization sessions. The findings indicated that the specific posterior-anterior passive accessory mobilization of the TMj significantly reduced pressure-induced pain in patients with OA in their dominant hand, measured by mechanical pressure algometer. However, according to the authors, this treatment did not lead to increased pinch or grip strength.

Physical Therapy

Laser therapy (class IV) is reported in the literature for the treatment of RA, but this is only a study project [80]. In this work, it is recommended to use the dosage and frequency indicated in the guidelines of the World Association of Laser Therapy [81]. Research has been published on the use of shock waves compared to hyaluronic acid infiltration [82]. A significant improvement in strength is observed in both groups, but the shock waves group shows better results on the pinch test since the end of treatment and for at least six months. To the authors' knowledge, only one paper reports ultrasound treatment combined with manual therapy. Even in this case, however, it is only a study project [83].

Infiltrative Therapy

For intra-articular injection of the TMj, hyaluronic acid or steroids are usually used. These treatments produce good results for pain and hand function. Injections with hyaluronic acid, however, show a longer-lasting effect and better results [84,85]. More recently, the use of platelet-rich plasma has been proposed in the literature [86].

A 2009 systematic review updated in 2017 [87] attempted to investigate the outcomes of conservative treatment of RA by considering a total of 95 randomized trials evaluating various pharmacological and non-pharmacological therapies. The findings report that, overall, the methodological quality of these randomized trials has improved since the last update, with more studies describing their methods of randomization and allocation concealment. However, randomized trials continue to be weakened by the lack of consistent case definitions and the lack of standardized outcome assessments specific to hand OA. The number and location of hand joints assessed continue to be underestimated, and only 25% of RCTs adequately described the method used to ensure allocation concealment. A meta-analysis could not be performed due to the marked heterogeneity of the studies, the insufficient statistical data available in the published RCTs, and the limited number of identical comparators. Hand OA is a complex area in which to study the effectiveness of therapies. Further large randomized trials with a more robust and specific methodological approach for hand OA are needed to draw clinically relevant conclusions on the efficacy of the different treatment options available.

Surgical therapy

Surgical treatment of RA is considered when medical treatment is not effective in pain relief. Surgical procedures vary according to the patient and his degree of OA. Therefore, the severity of joint involvement will be assessed by its clinical impact and its radiographic aspect using classifications such as that of Eaton and Glickel [49]. The involvement of other adjacent joints will also be decisive, as well as any deformities of the thumb that will limit the expected benefits.

A review identified 11 categories of surgical techniques [88]. Evidence for the effectiveness of these techniques is low. Although due to low evidence, they may be considered unreliable, it should be kept in mind that generating robust evidence in the field of surgery is almost impossible because of the numerous obstacles in conducting high-quality RCTs (e.g., inability to blind surgeons, urgent situations, and learning curves or patients' reluctance to participate in a study or to randomization). Four main techniques are used: arthrodesis, arthroplasty, trapeziectomy, and prosthesis.

Arthrodesis

Trapezium-metacarpal arthrodesis consists of the fusion of the trapezius and the first metacarpal, thus causing joint stability and disappearance of pain, at the expense of prolonged immobilization. A retrospective study by Forseth et al. in 2003 compared arthrodesis using plate and screw fixation with a Kirschner wire fixation assembly [89]. Forseth et al. showed that although the Kirschner wire and plate-screw fixation had comparable non-consolidation rates (approximately 8%), the plate-screw fixation group had a lower satisfaction rate. Arthrodesis is associated with good functional outcomes and low to moderate patient-reported disability and pain scores, but a high complication rate [90]. This technique is considered only in a few cases since other surgical techniques have better results, such as total or partial trapeziectomy and arthroplasty with interposition of cartilage or tendons.

Arthroplasty

The reference method in the international literature is the reconstruction of the anterior oblique ligament (or volar ligament) with arthroplasty through the interposition of a tendon from the radial flexor of the carpus described by Burton and Pellegrini in 1986 [91].

Trapeziectomy

Several reviews of the literature state that isolated trapeziectomy provides an analgesic and functional benefit equivalent to arthroplasty with ligament transposition and with fewer post-surgical complications [2,92]. Trapeziectomy (surgery in which the trapezius is removed) is effective on pain but at the cost of a decrease in grip strength and, for several months, instability of the thumb column [2]. Therefore, it is considered mainly for severe forms, i.e., Eaton and Glickel stages III and IV [93,94]. When adjacent joints are affected, only trapeziectomy can be used because other techniques would be ineffective on the pain produced by other joints. Radiological monitoring allows observing the progressive filling of the void and monitoring the occurrence of ossification or osteolysis of the area [95].

Prosthesis

The technique most used in the elderly is the use of prostheses to replace the upper extremity of the first metacarpus. There are many types of prostheses, but in any case, the trapezius bone must be sufficiently large and preserved. The expected benefits from the prostheses are reduction of deformity, functional recovery in three weeks, and recovery of grip strength in two months. However, prostheses lead to dislocations and loosening in about 15% of cases [95].

To our knowledge, only two studies have taken into consideration the costs and benefits, through a follow-up of up to 12 months, of conservative treatment alone, trapeziectomy alone, and trapeziectomy with ligament reconstruction and tendon interposition (LRTI). The study of Yoon et al. reported that [96] for the base case scenario, LRTI had the highest total cost of $8,798 whereas the cost of trapeziectomy was slightly lower at $8,251. Conservative management was modeled to have a $0 total expenditure because the frequency of clinic follow-up was assumed to be equivalent to postoperative clinic follow-up visits after either procedure. The average utility increase after trapeziectomy and trapeziectomy with LRTI is based on the study by Lane et al. [97]. Corresponding lifetime quality-adjusted life years (QALYs) were higher for the trapeziectomy group (14.14) compared to the LRTI group (13.80), and the conservative management group (11.43). Compared to conservative management, both trapeziectomy and LRTI were cost-effective treatments with an incremental cost-effectiveness ratio (ICER) of $3,045/QALY and $3,711/QALY, respectively. When comparing trapeziectomy to LRTI, LRTI was dominated by trapeziectomy because trapeziectomy cost less and scored more QALYs.

## Conclusions

RA is an arthritic degenerative process that affects the first joint of the thumb, i.e., the one between the trapezium bone and the base of the first metacarpus. RA manifests as pain at the base of the thumb, limiting grip strength and hindering everyday tasks. Pain initially occurs during specific movements but can progress to constant discomfort, joint stiffness, muscle atrophy, and severe deformities, like the "Z thumb," which seriously impact daily life. Prevalent in females, especially post-menopause, and linked to age, RA involves ligament and muscle structures, with causes ranging from hormonal influences to mechanical factors. Understanding the biomechanics, stability, and factors contributing to RA is crucial for effective intervention.

Pain assessment, joint mobility examination, and palpation are crucial for a correct differential diagnosis. Radiographic examination reveals joint space degeneration and osteophytes and helps classify RA stages. The studies on conservative approaches, such as multimodal intervention consisting of joint mobilization, neural mobilization, and exercise, are beneficial in reducing pain in patients with RA. When conservative therapy fails, surgical intervention is indicated. This study explores the role of ligaments, muscles, and anatomical variants in thumb joint degeneration, emphasizing the importance of stability and congruence, to provide therapists with an overview of the core issues related to the treatment of TMj OA.
